# Analysis of peripheral blood B and Tfh cells as predictors of antibody responses in individuals receiving candidate blood-stage malaria vaccines in a Phase Ia clinical trial

**DOI:** 10.1186/1475-2875-13-S1-P28

**Published:** 2014-09-22

**Authors:** Sean Elias, Kathryn Milne, Cecilia Chui, Susanne Hodgson, Persephone Borrow, Simon Draper

**Affiliations:** 1 Jenner Institute, University of Oxford, Oxford, UK; 2 Nuffield Dept of Medicine, University of Oxford, Oxford, UK

## Background

Here we report on a Phase Ia clinical vaccine trial using the AMA1 antigen from the blood-stage *Plasmodium falciparum* malaria parasite. A variety of promising ‘mixed-modality’ regimens were tested, with all volunteers primed with chimpanzee adenovirus-ChAd63, and then subsequently boosted with the orthopoxvirus MVA and/or protein-in-adjuvant (Alhydrogel ± CPG 7909) using 8 or 16 week prime-boost intervals. The induction of high titre, neutralizing antibodies is deemed essential for protection against blood-stage malaria. Peripheral blood antibody secreting cells (ASC) and memory B cells (mBC) play important roles in antibody production and maintenance. Lymph node (LN) CD4+ follicular helper T cells (Tfh cells) mediate cognate control of antigen-specific antibody responses. Recently Tfh-like cells have been found to be present in the CXCR5+ subset of memory CD4+ T cells in human peripheral blood. This facilitates analysis of the role of Tfh responses in regulation of vaccine-elicited antibody responses, although there is much debate over the relationship between LN and circulating populations. Phenotypic definition of the latter has also proved complex, with recent studies indicating that phenotypically-distinct Tfh populations with different functional profiles are elicited by different antigenic stimuli. Characterizing T cell and B cell populations across a variety of immunisation regimes may lead to greater understanding of their value as quantitative and qualitative predictors of antibody responses.

## Materials and methods

ASC and mBC responses were measured using a standardized B cell ELISPOT method, using fresh and frozen PBMC respectively, whilst antibody titre was measured using a standardized ELISA for AMA1. Multiparameter flow cytometry was also subsequently run using frozen PBMC to characterise total peripheral CD4+ Tfh populations and the AMA1-specific CD4+ T cell response.

## Results

AMA1 specific ASC, mBC and antibody responses followed classical kinetics at a level comparable to those previously described, with ASC and antibody responses strongly correlating. Notably, inclusion of CPG 7909 tended to lead to earlier detection of peripheral ASC responses at day 4. The relationship between baseline Tfh populations previously described as important in other infections/vaccine regimens, as well as AMA1-specific Tfh-like cells identifiable at post-boost time-points and ASC/mBC/antibody responses is currently being investigated.

## Conclusions

Very few significant differences were observed between groups with respect to ASC, mBC and antibody responses. Responses primed with ChAd63 appear to boost comparably whether or not CPG 7909 is included with the Alhydrogel adjuvant, though boosting with MVA trended towards lower responses.

